# Liquefaction optimization of grape pulp using response surface methodology for biopolyol production and bio-based polyurethane foam synthesis

**DOI:** 10.55730/1300-0527.3680

**Published:** 2024-03-18

**Authors:** Furkan ÇOLAKOĞLU, Emre AKDOĞAN, Murat ERDEM

**Affiliations:** Department of Chemistry, Faculty of Science, Eskişehir Technical University, Eskişehir, Turkiye

**Keywords:** Hardaliye, grape pulp, biopolyol, bio-based rigid polyurethane foam, experimental design

## Abstract

Both environmental and economic disadvantages of using petroleum-based products have been forcing researchers to work on environmentally friendly, sustainable, and economical alternatives. The purpose of this study is to optimize the solvothermal liquefaction process of grape pomace using response surface methodology coupled with a central composite design. After investigating the physicochemical properties of the liquified products (biopolyol) in detail, a bio-based rigid polyurethane foam (RPUF) was synthesized and characterized. The hydroxyl and acid numbers and viscosity values of all the biopolyols were analyzed. According to variance analysis results (%95 confidence range), both the reaction temperature and catalyst loading were determined as significant parameters on the liquefaction yield (LY). The model was validated experimentally in the following reaction conditions: 4.25% catalyst loading, 50 min reaction time, and 165 °C reaction temperature, which yields an LY of 81.3%. The biopolyols produced by the validation experiment display similar characteristics (hydroxyl number: 470.5 mg KOH/g; acid number: 2.31 mg KOH/g; viscosity: 1785 cP at 25 °C) to those of commercial polyols widely preferred in the production of polyurethane foam. The physicochemical properties of bio-based foam obtained from the biopolyol were determined and the thermal conductivity, closed-cell content, apparent density, and compressive strength values of bio-based RPUF were 31.3 mW/m·K, 71.1%, 33.4 kg/m^3^, and 105.3 kPa, respectively.

## 1. Introduction

The use of petroleum as an energy source harms the natural environment. These damages are increasing over time due to the rising global energy consumption driven by the growing world population. Consequently, researchers are seeking alternative, environmentally friendly, and sustainable products. The use of industrial and agricultural biomass residues as bio-based raw materials holds great potential as renewable energy sources. Biomass is abundant, accessible, and renewable resources that can easily be converted into different types of high-value materials. Agricultural biomass, produced by photosynthesis by using carbon dioxide (CO_2_) in the atmosphere, has a shorter generation time, unlike fossil fuels. Additionally, biomass is considered a “carbon neutral” fuel because the CO_2_ released during its combustion is roughly equal to the amount it absorbed from the atmosphere during its growth [1].

To evaluate biomass, conversion into valuable products can be achieved through various liquefaction methods the appropriate reaction routes. Acid-catalyzed solvothermal liquefaction (ACSL) is the most commonly used method for liquefying biomass to produce one of the components needed for the formation of polyurethane foam. In ACSL processes, biomass is typically liquefied using a mixture of polyhydric alcohols as the solvent. The amount of catalyst, reaction temperature, and reaction time are variable factors in these processes [2,3]. Several researchers have lately produced biopolyols derived from the liquefaction of various lignocellulosic biomass sources, such as spent coffee grounds [4], cork powder [5], cotton stalk [2], banana bamboos [6], cotton burrs [7], wheat straw [8], and peanut shells [9], to create bio-based RPUFs.

Grapes are one of the largest fruit crops in Türkiye. According to the Ministry of Agriculture and Forestry of the Republic of Türkiye, 4.1 million tons of grapes were produced in Türkiye over an area of 420,000 hectares during the 2020 production period^1^ . About 60% of the world’s grape production is used to make wine and similar beverages, generating a large amount of grape pomace as a byproduct. Grape pomace consists of grape skins, stems, and seeds. It contains cellulose, hemicellulose, lignin, protein, and phenolic compounds. The higher lignocellulose content can have a positive effect on the utilization of grape pomace [10]. Shao et al. optimized four liquefaction factors and successfully liquefied grape seeds in the mixed solvent of polyethylene glycol 400 and glycerol for biopolyol production [11]. However, research on the liquefaction of GS to produce biopolyol and polyurethane foam is rarely reported.

Polyurethanes are high-performance polymers formed by polycondensation reactions of polyols and diisocyanates. Polyurethanes are frequently used in the manufacture of thermoplastics, elastomers, foams, paints, adhesives, and rubber. Rigid polyurethane foam (RPUF) is one of the most widely used types of polyurethane. Heat and sound insulation are important application areas of RPUFs [12]. The types of isocyanates and polyols used in the RPUF formulation greatly affect the properties of foams. In addition, the massive use of commercial polyols for RPUF synthesis poses various economic and environmental problems. In this context, due to increasing concerns associated with petroleum use, biopolyols produced from grape pomace (GP) via liquefaction technique present a compelling alternative to commercial petroleum-based polyols [3].

There are numerous studies on the conversion of different agricultural crop residues into valuable biopolyols; however, to the best of our knowledge, this is the first study to evaluate GP via the ACSL using experimental design for biopolyol and polyurethane production. In the present study, solvothermal liquefaction of GP was performed by using 20 wt% glycerol and 80 wt% PEG400 solvent mixture in the presence of sulfuric acid as a catalyst. The effects of the reaction time, reaction temperature, and catalyst loading on the LY were investigated to optimize LY using response surface methodology (RSM) coupled with a central composite design (CCD). Finally, RPUF was produced using a one-shot method with half substation of GP-based biopolyol with the commercial polyol. The produced bio-based RPUF was characterized via SEM, FT-IR spectroscopy, gas displacement pycnometer, universal test machine, and heat flow meter.

## 2. Materials and methods

### 2.1. Materials

GP was provided by Kırklareli Rumeli Hardaliyesi Factory (Kırklareli, Türkiye). Polyethylene glycol 400 (PEG 400) and glycerol (G) were purchased from Tekkim Kimya (İstanbul, Türkiye). Sulfuric acid (SA), 1,4-dioxane, sodium hydroxide (NaOH), phthalic anhydride pyridine, imidazole, ethyl alcohol, toluene, acetone, potassium hydrogen phthalate, silicon oil (Rhodorsil Oils 47), tetramethyl ethylenediamine, dibutyltin dilaurate, and barium chloride were reagent grade and purchased from Sigma-Aldrich (Missouri, USA). polymethylene diphenyl isocyanate (pMDI), surfactant (Tegostab B 8476), and petroleum-based polyol were provided by Ravago Petrokimya Üretim A.Ş (İstanbul, Türkiye). The distilled water, used as a chemical blowing agent, was produced in our laboratory.

### 2.2. Methods

#### 2.2.1. Pretreatment of GP

After drying the GP residues at 70 °C until they reached a constant weight in an oven, the residues were ground and sieved below 250 μm.

#### 2.2.2. Solvothermal liquefaction of GP

The ACSL method was carried out in accordance with the literature [13]. The liquefaction reaction was stirred in a 250-mL reaction flask with an overhead stirrer. The mixture of liquefying solvent was formed with 20 wt% glycerol and 80 wt% PEG400 and the liquefying solvent/GP ratio was fixed at 4/1 (wt/wt). The amount of sulfuric acid (SA) was adjusted according to the amount of liquefaction solvent mixture. Before starting the reaction, GP powder (5.0 g) and liquefying solvent (20g) were mixed for 3 min and then placed in a preheated silicon oil bath. After the reaction mixture reached the desired temperature (150–180 °C), SA was immediately poured into the flask. The reaction was continued for the specified times at 200 rpm. After the reaction time (20–100 min) was over, the mixture was transferred to a cold-water bath to quench the reaction. The mixture was then diluted with a solution of 100 ml, containing 80 mL of 1,4-dioxane and 20 mL of water, and stirred continuously for 1 h. The diluted mixture was filtered via suction filtration to isolate the residue. The insoluble residue was dried until it reached the constant weight in a laboratory oven at 105 °C. The solvent was evaporated using a rotary evaporator to obtain biopolyol. The LY was calculated as Equation 1.


(1) $$LY(\%)=\frac{Y_{2}\times 100}{Y_{1}}$$

#### 2.2.3. Experimental design and process optimization

It is very important to detect the variability of the parameters affecting the LY to increase efficiency with fewer experimental studies. At this point, CCD of RSM is one of the preferred applications to study the effects of different variables on the targeted response.

Minitab 19 statistical software was used to determine the effects of reaction parameters such as catalyst loading (X_1_), reaction time (X_2_), and reaction temperature (X_3_) on the LY (Table 1). The effects of the three independent variables on LY, along with the experimental design matrices containing both and the experimental design matrices with coded and real values corresponding to the results of LY, are presented in Table 2. A total of 17 experiments were performed to obtain both biopolyols and the LY values. The relationships between the obtained experimental and predicted data were analyzed with a 95% confidence level analysis of variance. The accuracy and reliability of the created model were examined with the correlation coefficient (R^2^). Minitab software was used to visualize response surface and contour plots.

#### 2.2.4. Characterization of the biopolyols

The hydroxyl and acid numbers of biopolyols were determined by ASTM D 4274 (Method D) and ASTM D 4662 test standards, respectively. Viscosities of biopolyols at 25 °C were determined with a Brookfield DV3T Viscometer (AMETEK Brookfield, Middleboro, USA), following the ASTM D 4878-08 standard. The chemical structures of the biopolyols were analyzed using the ATR mode of the FT-IR spectrometer (Perkin Elmer, Massachusetts, USA) over a scan range of 4000–700 cm^−1^. Molecular weight analysis of biopolyol was performed using gel permeation chromatography (Agilent Technology, Santa Clara, USA) equipped with a refractive index detector (G1362A) and a GPC column (TSK G3000 PWXL). Tetrahydrofuran was used as the mobile phase at a flow rate of 1.0 mL/min and column temperature of 35 °C under 10.0 MPa pressure. The average molecular weight of the samples was calculated using a calibration curve of monodisperse polystyrene standards.

#### 2.2.5. Preparation of RPUF

RPUFs were fabricated with a one-shot and free-foaming method. The bio-based RPUF was synthesized using the biopolyol produced from the optimum ACSL reaction condition, with petroleum-based polyol partially substituted with biopolyol in a 1:1 ratio (Table 3). The polyol mixture, containing polyols, water, catalysts, and surfactant, was placed in a 500-mL syringe in an appropriate amount and mixed using a mechanical stirrer at 2000 rpm for 2 min. Upon completion of mixing, pMDI was swiftly added to the mixture using another syringe while stirring continued. After the foam mixture reached the creamy phase (5–8 s), it was rapidly poured into a metal mold with dimensions of 21 cm × 21 cm × 7 cm (length, width, thickness). Once the foam formation was complete, it was removed from the mold and left to cure for 3 days.

#### 2.2.6. Characterization of RPUF

The thermal conductivity of the foams, cut into dimensions of 20 cm × 20 cm × 3 cm, was measured using the Linseis HFM 300 Heat Flow Meter following the ASTM C518 standard. While the temperature of the upper plate was adjusted to 38 °C, the lower one was adjusted to 10 °C, keeping the average foam temperature at 24 °C.

To determine their closed cell contents and apparent densities, three pieces of the foams, each measuring 3 cm × 3 cm × 3 cm, were used. The final dimensions of each piece were measured with a caliper to calculate the apparent density. The closed cell contents were then determined using a gas displacement pycnometer (Micromeritics AccuPyc II 1340) according to ASTM D-6226. The average value of the results was reported.

The compressive strength values were determined using three pieces of foam, each cut into dimensions of 5 cm × 5 cm × 3 cm, using the universal testing machine (Zwick/Roell brand Proline) according to ASTM D-1621 test standard at room temperature. Compressive stress at 10% strain was applied to the foams, and the strength values were measured at this deformation. The average values of the results were recorded.

Morphological characterization of RPUFs was performed employing scanning electron microscopy (SEM, Carl Zeiss Ultra Plus, Oberkochen, Germany) using an appropriate voltage at 100× magnification. The sliced foam pieces were coated with gold to have a conductive layer.

## 3. Results and discussion

### 3.1. Statistical analysis

#### 3.1.1. Experimental design

In the presented study, the process of obtaining biopolyol from GP by ACSL was optimized to increase the percentage of LY based on 3 different experimental parameters to prevent both economic and time loss. Table 2 displays the experimental CCD matrix for the production of biopolyols along with the corresponding responses. It was detected that LY values diversified between 59.35% and 88.99% according to the liquefaction reaction conditions. When the reaction conditions are 180 °C, 60 min, and 4 wt% catalyst loading, the highest LY value (86.17%) was achieved. This result revealed that ACSL is an efficient and feasible way to convert GP to biopolyol. A quadratic polynomial equation was formulated to express the correlation between LY and reaction parameters in terms of coded values from the model. The relevant equation is presented in Equation 2.


(2) $$Y_{LY}=77.15+5.27X_{1}+2.50X_{2}+4.81X_{3}-1.51X_{1}^{2}+0.14X_{1}X_{3}-1.48X_{2}X_{3}$$

where Y_LY_ is the liquefaction yield (%), X_1_ is the catalyst loading, X_2_ is the reaction time (min), and X_3_ is the reaction temperature (°C). The positive coefficients cause the LY values to increase, whereas the negative coefficients cause them to decrease in the studied range. While the model was created, incorrect responses can be obtained due to operational errors. These values can be considered outliers and they can be extracted from the model to better analyze the experimental results; therefore, to obtain a more robust model, experiment number 2 was detected as an outlier and removed from the model.

The variance analysis (ANOVA) results of the obtained quadratic model were tabulated in Table 4. According to the F-test, the F value of the quadratic model was detected at 104.55 with a low probability value (p-value <0.001). This result indicates that the obtained model is quite significant. A p-value is used as a measure of the statistical significance of research results and when the p-value is lower than 0.05, the research results are generally considered to be statistically significant. In this case, the model terms of X_1_, X_2_, X_3_, X_1_^2^, X_1_X_3_, and X_2_X_3_ were significant. The remaining terms were not considered significant because p-values were greater than 0.005. The p-value of the lack of fit was found to be 0.450, indicating a lack of statistical significance. This implies that the experimental error at the center point (experiment numbers 9, 10, and 17) of the model falls within an acceptable range, validating the credibility of the predicted results [14]. Furthermore, the regression coefficient was calculated to be 0.9859, which is close to 1. It is also implied that the experimental data for the liquefaction reactions fitted well with the predicted value of the model [15].

#### 3.1.2. Optimization of ACSL parameters

**Figure 1:**
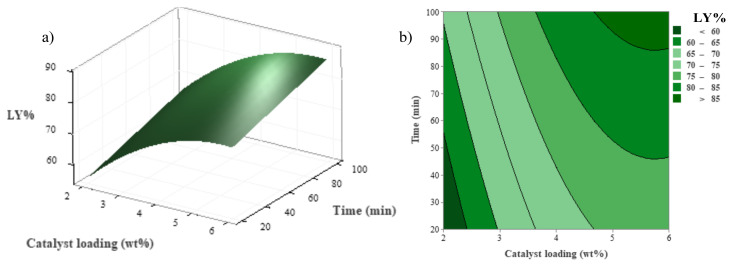
Combined effect of catalyst loading and reaction time on LY at constant temperature (T = 160 °C); a) 3D surface, b) contour plots.

Contour and three-dimensional (3D) surface plots were drawn using Minitab software based on the quadratic regression equation and are depicted in Figures 1 and 3. During the examination of the combined effects of two specific factors, the remaining factor was kept constant at the center point (0 level in Table 1) in these figures.

Figures 1a and 1b illustrate 3D response surface and contour plots depicting the effects of catalyst loading and reaction time on the liquefaction yield. LY reached its maximum value in the range of 5%–6% catalyst loading and 60–100 min of reaction time. Beyond those critical points, the LY showed a tendency to decrease. The recondensation reaction in the depolymerized lignocellulosic content of GP might be the reason behind this decrease in the LY value, as reported by other researchers [16–18]. According to Figure 1b, to achieve an LY value of 85% and above, it is advisable to establish reaction conditions where the reaction time and catalyst loading are both greater than 86.1 min and 4.7%, respectively.

**Figure 2:**
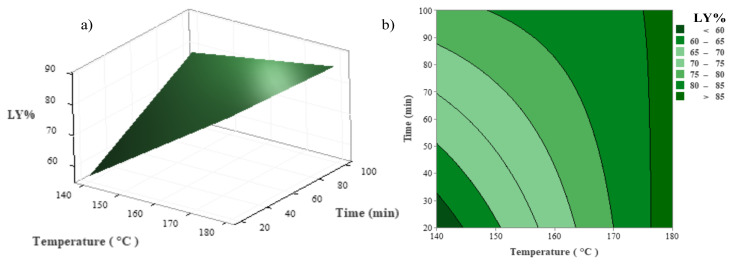
Combined effect of reaction temperature and reaction time on LY at a constant amount of catalyst (4%); a) 3D surface, b) contour plots.

**Figure 3:**
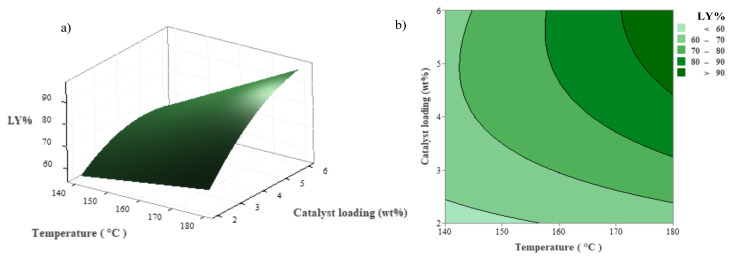
Combined effect of reaction temperature and catalyst loading on LY at a constant reaction time (60 min); a) 3D surface, b) contour plots.

Figures 2a and 2b show the combined effects of reaction time and temperature on LY values. It is observed that the effect of reaction temperature has a greater influence than the reaction time in enhancing the LY value. The conversion of GP increased to 86.43% when the reaction temperature was boosted from 140 °C to 180 °C. To reach the LY value of more than 85% at the minimum reaction time (for example 20 min), the reaction temperature should be at least 176.4 °C. Furthermore, when Figures 3a and 3b are examined, it is evident that an increase in catalyst loading enhances the LY value. Additionally, to achieve an LY value of 90%, it is necessary for the reaction temperature and catalyst loading to be greater than 171 °C and 4.4%, respectively.

As a result, the optimal reaction conditions were estimated for converting GP into biopolyol were estimated using Minitab software. The optimal ACSL reaction parameters were identified as follows: 4.25% catalyst loading, 50 min reaction time, and 165 °C reaction temperature, yielding an LY value of 80.1%. These optimal values were selected in the present study to minimize energy and time consumption while ensuring that the obtained biopolyols exhibit desirable properties for polyurethane production.

#### 3.1.3. Validation of the model

In order to validate the model, the validation experiments on the optimal reaction conditions were performed three times with the same procedure, and Table 5 illustrates the results of the experiments. While the experimental LY value was 81.3% ± 1.5, the LY value for the selected target estimate was 80.1%. The error was found to be less than 1.5% which indicates that the proposed model was in excellent agreement with those predicted reaction conditions. As a result, the obtained model was highly accurate and reproducible.

### 3.2. Physicochemical properties of obtained biopolyols

The goal of this study was to convert GP formed after the production of hardaliye into biopolyols according to the concept of biorefinery. It was demonstrated that GP, annually generated as a byproduct of the beverage industry, holds significant potential for conversion into biopolyols, essential components of polyurethane. Moreover, exploring the properties of biopolyols is crucial to assess their viability for use in polyurethane production. To that end, their hydroxyl and acid numbers, and viscosity values were evaluated. In 2016, Shao et al. evaluated the feasibility of liquefying grape seeds to produce biopolyol [11]. In contrast, our study focused on liquefying grape pulp. In addition to seeds, pulp also contains other components of the grape herb/fruit. Although both biomasses are similar, they have chemical distinctions. As a result, their behavior during the liquefaction process varies. Consequently, it is expected that the properties of these biopolyols obtained from different studies will differ from each other.

#### 3.2.1. Hydroxyl number

The physicomechanical properties of polyurethanes can be arranged by the changes in the hydroxyl number of the polyols. In the process of liquefying biomass, liquefaction solvents are essential. These solvents, which are also polyols, must not only facilitate efficient and rapid liquefaction of biomass but also have the appropriate polyol properties for the intended PU applications. In this study, while the hydroxyl number of liquefying solvent (PEG400/G weight ratio of 4/1) was 632.6 mg KOH/g. In comparison, the hydroxyl number of the control experiment, conducted at the center point (0 level in Table 1) using the liquefying solvent without GP powder, was determined to be 406.2 mg KOH/g. The reduction in the hydroxyl group can be related to the dehydration of water molecules [5].

**Figure 4:**
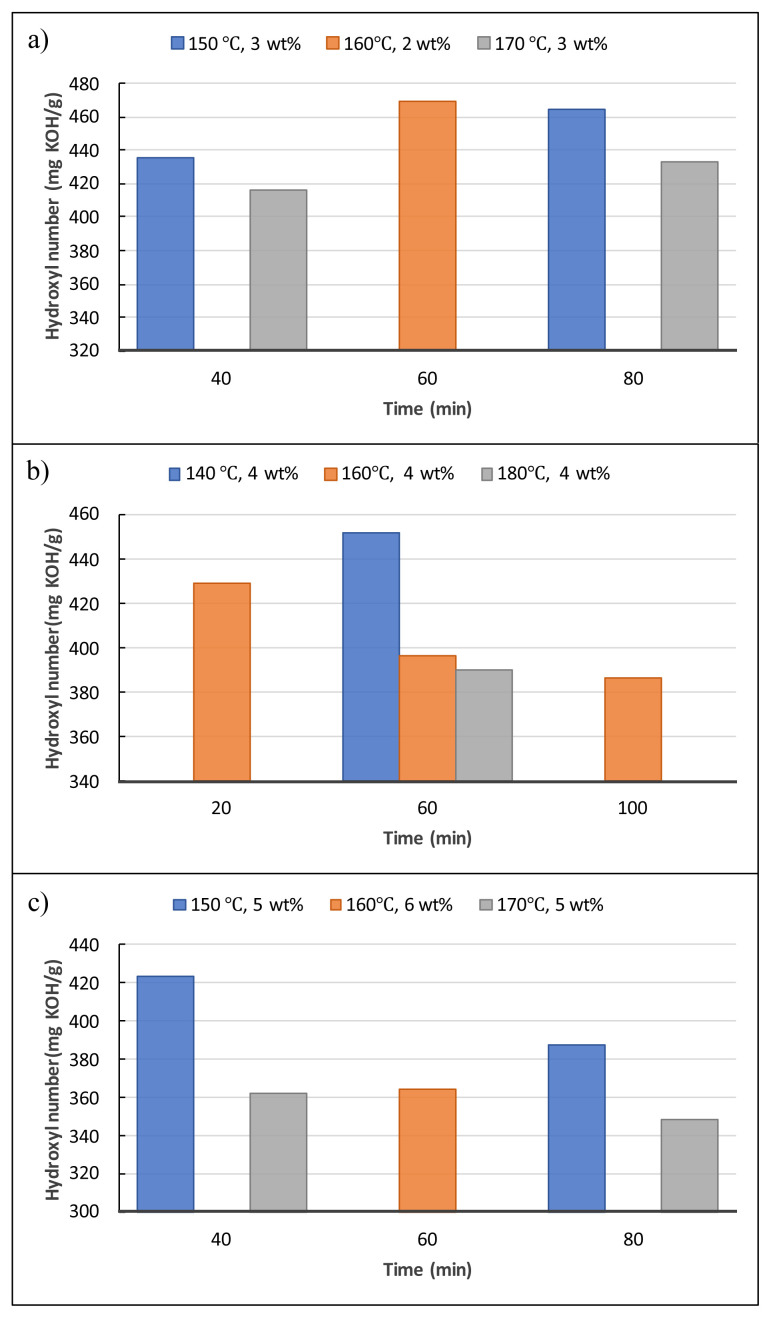
Effects of liquefaction reaction parameters on the hydroxyl numbers.

Figure 4 depicts the influences of the liquefaction reaction parameters on the hydroxyl numbers of the obtained biopolyols. The reaction time and temperature were the determinant parameters for the reactions carried out at 3 %wt of catalyst (SA). For instance, when the reaction temperature was raised from 150 °C to 170 °C for 40-min reactions, the hydroxyl numbers of biopolyols started to decline. However, when the time was increased from 40 min to 80 min for both 150 °C and 170 °C reactions, the increases in the hydroxyl number were observed (Figure 4a). The highest hydroxyl number, 469.74 mg KOH/g, was detected as the reaction parameters were 2 wt% SA, 60 min, and 160 °C. The hydroxyl numbers decreased as the reaction time increased gradually (20, 60, and 100 min) at the parameters of 4 wt% SA and 160 °C. Furthermore, when the reaction temperatures were increased for 60-min reactions, significant decreases in the hydroxyl numbers were observed (Figure 4b). As the catalyst loading was increased to 5 wt%, the opposite tendency in hydroxyl values to those of 3 wt% was observed. When the reaction time was increased from 40 min to 80 min for both 150 °C and 170 °C reactions, decreases in the hydroxyl number were observed (Figure 4c). It can be concluded from the results that all the reaction parameters have distinct influences on the hydroxyl numbers of biopolyols. The average hydroxyl number of biopolyols obtained from the validation parameters (4.25 wt% SA, 50 min, and 165 °C) was detected as 470.5 mg KOH/g. As a result, considering their hydroxyl numbers, all biopolyols obtained in this study are suitable raw material candidates to produce RPUFs, which are utilized in various sectors including sandwich panel production, the refrigeration industry, furniture, and automotive applications [19,20].

#### 3.2.2. Acid number

Another significant parameter affecting the polymerization reaction kinetics is the acid number of polyols. The nature of biopolyols synthesized via ACSL is acidic due to the acidic catalyst used in the method. Before fabricating RPUF, the acidity of biopolyol must be decreased using basic solutions of NaOH and Na_2_CO_3_ to obtain RPUFs with fine cellular structure [21,22]. Therefore, it is desirable for the acid number of biopolyol to be less than 5 mg KOH/g during the production of RPUF [19].

**Figure 5:**
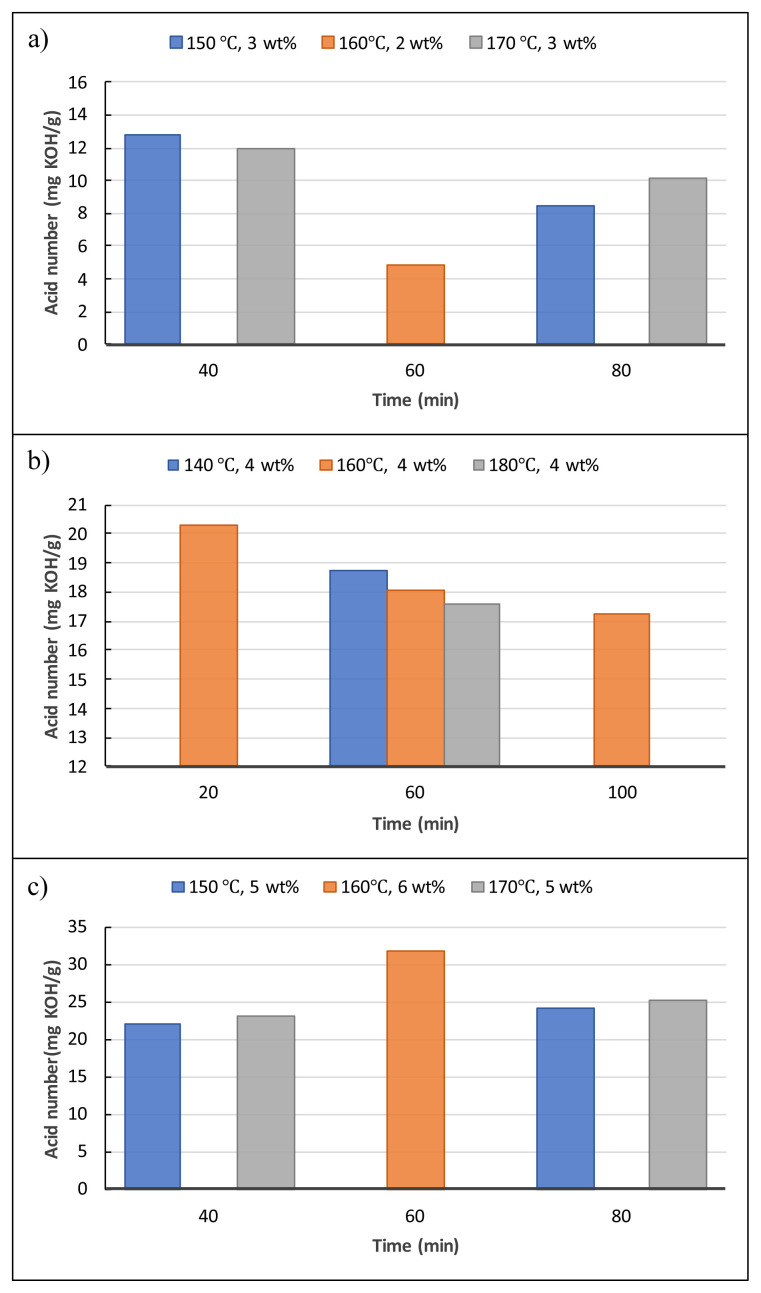
Effects of liquefaction reaction parameters on the acid numbers.

Figure 5 displays the influences of the liquefaction reaction parameters on the acid numbers of the obtained biopolyols. In this study, the acid number was found to be 32.11 mg KOH/g for the control experiment. As displayed in Figure 5a, when the reaction temperature was raised from 150 °C to 170 °C for 40 min and 3 wt% SA reactions, the acid numbers of biopolyols decreased. However, in the same temperature conditions for 80 min and 3 wt% SA reactions, the acid numbers of biopolyols increased. The acid number of biopolyol reached the lowest value (4.85 mg KOH/g) at the parameters of 2 wt% SA, 60 min, and 160 °C (Figure 5c). The acid numbers decreased as the reaction time increased gradually (20, 60, and 100 min) at the parameters of 4 wt% SA and 160 °C. The acid number of 31.75 mg KOH/g was the highest and it was detected as the reaction parameters were 6 wt% SA, 60 min, and 160 °C (Figure 5c). As expected, the catalyst loading was the most effective parameter for the acid numbers of biopolyols. The average acid number of the biopolyols produced from the validation experiments was determined as 23.85 mg KOH/g. These results were close to previously reported values for the acid numbers of biopolyols. [17,20,23]

#### 3.2.3. Viscosity

When the suitability of polyols is evaluated for the use of polyurethane foam production, viscosity emerges as another important parameter. Polyols with high viscosity obstruct mixing with RPUF components, thus negatively affecting the polymerization reaction.

**Figure 6:**
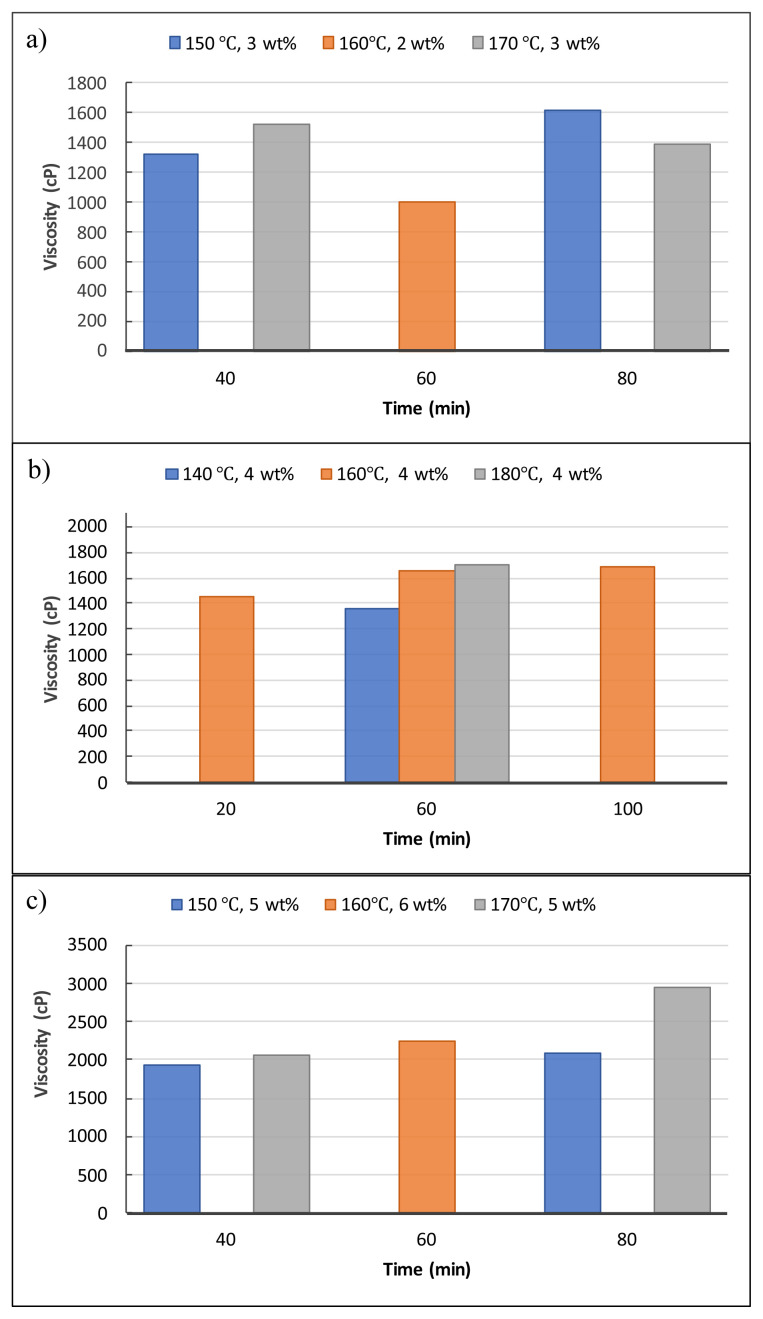
Effect of liquefaction reaction parameters on the viscosity values.

Figure 6 indicates the influences of liquefaction reaction parameters on the viscosity values of biopolyols. The viscosity was found to be 317.5 cP for the control experiment. As illustrated in Figure 6a, when the reaction temperature was raised from 150 °C to 170 °C for 40 min and 3 wt% SA reactions, the viscosity values of biopolyols increased, whereas, in the same temperature conditions for 80 min and 3 wt% SA reactions, the viscosity values of biopolyols declined. The viscosity value caused a reduction for the parameters of 2 wt% SA, 160 °C, and 60 min reaction (Figure 6a). The viscosity values of the biopolyols were raised from 1445 cP to 1685 cP as the reaction time was raised from 20 min to 100 min at the parameters of 4 wt% SA and 160 °C. The viscosity values of the biopolyols raised from 1358 cP to 1703 cP as the reaction temperature was raised from 140 °C to 180 °C for the 60-min reactions (Figure 6b). When the reaction temperature was increased from 150 °C to 170 °C in the presence of 5 wt% SA, a slight increase was detected for the reaction time of 40 min, while a significant increase was observed for the reaction time of 80 min. Furthermore, the increase in the reaction time from 40 min to 80 min also increased the viscosity values under the same reaction parameters (Figure 6c). The average viscosity value of the biopolyol produced from the validation experiments was determined as 1785 cP. In conclusion, considering the viscosity values, all the obtained biopolyols can be deemed suitable for use in the polyurethane foam industry.

### 3.3. FTIR and GPC analysis

**Figure 7:**
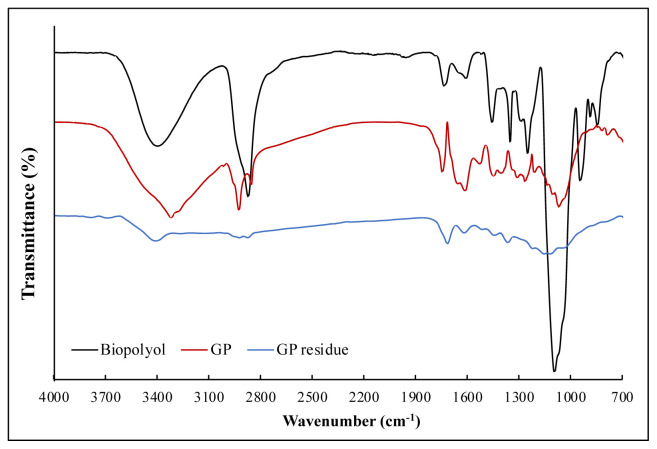
FT-IR spectra of GP, GP residues, and biopolyol.

FT-IR spectra of GP, GP residue, and biopolyol synthesized in the validation experiment are presented in Figure 7. Several similar peaks are seen in the spectra of GP and GP residue because of the functional groups present in the lignocellulosic structure. However, significant reductions were observed in peak intensities for the GP residue. It can be concluded that the macromolecules of GP were broken down by ACSL and then liquefied [6]. The strong peak at 3406 cm^−1^ was associated with the hydroxyl (–OH) stretching vibrations. The increase in vibration peak intensity also indicated the presence of many hydroxyl groups. The lignocellulosic content of GP was probably the source of hydroxyl groups [24]. The peak at 2855 cm^−1^ of biopolyol for the C-H stretching of CH_3_, CH_2_, and CH groups shifted and increased, indicating that the relevant bonds stretching between the wavelengths of 3050 cm^−1^ and 2850 cm^−1^ in GP were rearranged by liquefaction [11]. The peaks at 1725 cm^−1^, 1650 cm^−1^, and 1470 cm^−1^ for biopolyol were attributed to the C=O stretching (the presence of liquefied structures of ester and acetyl groups in hemicellulose), aromatic C=C stretching, and aromatic C-H in-plane bending vibrations. Although their intensity varies, these absorption bands were observed at approximately the same wavelengths for GP and GP residue. Additionally, the peak intensity at 1160 cm^−1^ for biopolyol increased, indicating the formation/increase of the number of ether bonds during the liquefaction reaction [19]. The sharp peak at 1091 cm^−1^ for biopolyol was also associated with the -C-O-C- stretching vibrations.

**Figure 8:**
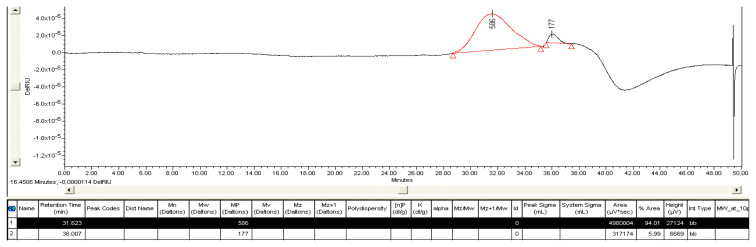
GPC chromatogram of biopolyol.

The GPC chromatogram of the biopolyol is presented in Figure 8. The data of GPC analysis indicated that the liquefaction mixture comprised of two major fractions with different molecular weights. The ratio of the peak areas to the total area was used to determine the percentage share of each of the individual peaks. Regarding these results, it was seen that the average molecular weight of synthesized polyol was 586 g mol^−1^. A molecular weight value of 5180 g mol^−1^ has been reported by Shao et al. for liquefied grape seed using similar experimental conditions (solvent mixture: PEG400 and glycerol with the ratio at 4:1; catalyst: sulfuric acid ranging from 1% to 6%; liquid–solid ratios ranging from 2 to 5; reaction temperature: 100–200 °C; reaction time, 40–320 min) [11]. Comparing these results, the biopolyol obtained in this study suggests shorter carbon chains within the liquefied grape seed structure.

### 3.4. Physicomechanical and morphological properties of foams

The properties of RPUFs are mainly influenced by their cellular morphology, type of polyols and isocyanates, the amount and type of additives, and the amount and type of blowing agent, etc. [25–28]. Some important properties of RPUFs are presented in Table 6.

**Figure 9:**
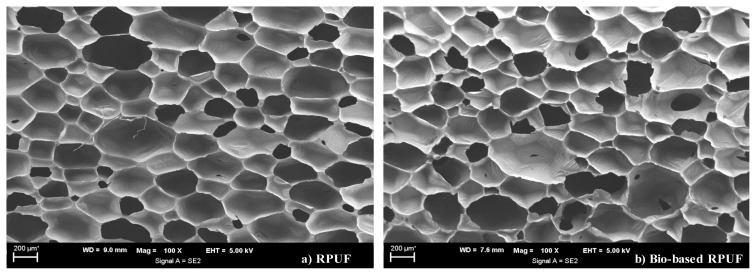
SEM images of foams; a) RPUF and b) bio-based RPUF.

Figure 9 shows the SEM images of RPUF and bio-based RPUF. It is seen that the foam cells exhibit a polyhedral shape, and there is no observable cell destruction with the introduction of biopolyol. Additionally, the average cell size of the bio-based RPUF closely resembles that of the standard RPUF. While the average cell size of the standard RPUF was 280 ± 75 μm, the cell size of the bio-based RPUF was 295 ± 95 μm.

The insulation performance of foams is mainly determined by the values of thermal conductivity. The thermal conductivity value of RPUF was 28.4 mW/m·K, whereas the thermal conductivity value of bio-based RPUF increased to 31.3 mW/m·K when the biopolyol was replaced with a petroleum-based polyol in half amount. The decrease in the closed-cell content probably caused the increase in the thermal conductivity value of the bio-based RPUF. While the closed-cell content of the bio-based RPUF was 71.1%, the same value for the reference RPUF was 88.8%. Nevertheless, the thermal conductivity value of the bio-based RPUF is higher than that of commercial polyurethane foam (20–24 mW/m·K); however, the thermal conductivity value found in this study is much lower than that of polystyrene foam, polyvinyl chloride foam, and polypropylene foam [29]. In conclusion, the produced bio-based RPUF is deemed suitable for use as a thermal insulation material.

The compressive strength value depends mostly on the apparent density of RPUFs. The higher the density, the higher the compressive strength value [30]. While the compressive strength value of RPUF was 210.8 kPa, the value for the bio-based RPUF decreased to 105.3 kPa. The reason for this reduction in the compressive strength value can be attributed to the decreasing density of bio-based RPUF. The presence of biopolyol is expected to negatively affect the compressive strength of polyurethane foam and this is commonly found in the literature [10,31–33]. Considering all the results, it can be concluded that the bio-based RPUF holds great potential as a sustainable insulation material.

## 4. Conclusion

The liquefaction of GP via the ACSL method was carried out in the presence of an acidic catalyst (SA) using PEG400/G as the liquefying solvent. CCD of RSM was used as a design of experiment technique to optimize the liquefaction reaction parameters. The resulting model revealed significant effects of the liquefaction reaction parameters on the LY. Based on variance analysis (ANOVA), the catalyst loading was the most significant parameter on the LY. The accuracy and reliability of the model were demonstrated with a high regression coefficient (R^2^) of 0.9859. In addition, the reliability was confirmed by the validation experiments and the absolute error was obtained as 1.48%. The biopolyol obtained from the validation experiments exhibited proper physicochemical properties to be used in the RPUF industry and had a hydroxyl number of 470.5 mg KOH/g, acid number of 2.31 mg KOH/g, and viscosity of 1785 cP at 25 °C. Using this biopolyol, a bio-based RPUF was synthesized by replacing petroleum-based polyol in half amount and its physicomechanical properties were determined. The values of thermal conductivity, closed-cell content, apparent density, and compressive strength were 31.3 mW/m·K, 71.1%, 33.4 kg/m^3^, and 105.3 kPa, respectively. As a result, ACSL can be preferred for the valorization of industrial residue GP based on biorefinery concepts, and the resulting biopolyol can be utilized in polyurethane formulations.

## Figures and Tables

**Table 1:** Experimental range and the levels of independent variables.

Independent variables	Symbol	Range and levels				

		−2 (α)	−1	0	1	2 (α)
Catalyst loading (wt%)	X_1_	2	3	4	5	6
Reaction time (min)	X_2_	20	40	60	80	100
Reaction temperature (°C)	X_3_	140	150	160	170	150

**Table 2:** Experimental CCD matrix for the production of biopolyols along with the corresponding responses.

Order	Independent variables						Response (experimental)	Response (predicted)

	Real values			Coded values				

	X_1_	X_2_	X_3_	X_1_	X_2_	X_3_	LY (%)	LY (%)
1	3	40	150	−1	−1	−1	64.4	62.9
2*	5	40	150	1	−1	−1	73.7	-
3	3	80	150	−1	1	−1	71.3	70.9
4	5	80	150	1	1	−1	79.1	78.6
5	3	40	170	−1	−1	1	74.7	72.8
6	5	40	170	1	−1	1	85.6	86.2
7	3	80	170	−1	1	1	74.7	74.9
8	5	80	170	1	1	1	89.0	88.2
9	4	60	160	0	0	0	76.4	77.2
10	4	60	160	0	0	0	77.0	77.2
11	2	60	160	−2	0	0	59.4	60.6
12	6	60	160	2	0	0	81.9	81.7
13	4	20	160	0	−2	0	70.8	72.1
14	4	100	160	0	2	0	81.5	82.2
15	4	60	140	0	0	−2	66.5	67.4
16	4	60	180	0	0	2	86.2	86.9
17	4	60	160	0	0	0	78.5	77.2

* This experiment was considered an outlier to obtain a more robust model.

**Table 3:** Formulation of biopolyol-based RPUF.

Components	Amounts (g)	

	RPUF	bio-based RPUF
Petroleum-based polyol	60	30
Biopolyol	-	30
pMDI	93.70	93.70
Water	1.8	1.8
Amine catalyst	0.42	0.42
Tin catalyst	0.12	0.12
Surfactant	1.2	1.2

**Table 4:** ANOVA of quadratic model for liquefaction yield.

Source	Model					Remarks

	DF	Adj SS	Adj MS	F-value	p-value	
Model	6	985.676	164.276	104.55	0.000	Significant
X_1_	3	395.697	305.525	194.44	0.000	Significant
X_2_	1	89.126	89.126	251.83	0.000	Significant
X_3_	1	336.166	336.166	56.72	0.000	Significant
X_1_^2^	1	56.721	56.721	213.94	0.000	Significant
X_2_^2^	1	1.197	1.197	0.83	0.398	
X_3_^2^	1	0.793	0.793	0.55	0.487	
X_1_X_2_	1	4.130	4.130	2.86	0.142	
X_1_X_3_	1	12.512	12.512	7.96	0.020	Significant
X_2_X_3_	1	14.148	14.128	8.99	0.015	Significant
Error	9	14.142	1.571			
Lack of fit	7	11.920	1.703	1.53	0.450	Not significant
Pure error	2	2.222	1.111			
Total	15	999.817		R^2^ = 0.9859, adjusted R^2^ = 0.9764, predicted R^2^ = 0.9426		

**Table 5:** Validation of the model.

Exp.	X_1_ (%)	X_2_ (min)	X_3_ (°C)	Liquefaction yield (%)	Error (%)
Predicted	4.25	50	165	80.1	1.48
Experimental				81.3 ± 1.5	

**Table 6:** Some important properties of RPUF and bio-based RPUF.

Sample	Thermal conductivity (mW/m·K)	Closed cell content (%)	Compressive strength (kPa)	Density (kg/m^3^)
Bio-based RFUF	31.3±0.22	71.1±3.1	105.3±7.8	31.2±2.9
RPUF	28.4±0.32	88.8±2.2	210.8±11.2	39.0±1.3

## Footnotes

1 Republic of Türkiye Ministry of Agriculture and Forestry, Agricultural Economic and Policy Development institute (2023). Tarım ürünleri piyasaları [online]. Website https://arastirma.tarimorman.gov.tr/tepge/Menu/27/Tarim-Urunleri-Piyasalari [accessed 16 July 2023]
